# Supplementary material for: A Facile Strategy toward the Preparation of a High-Performance Polyamide TFC Membrane with a CA/PVDF Support Layer
Source: Nanomaterials (Basel). 2022 Dec 19;12(24):4496. doi: 10.3390/nano12244496 (PMC9785465; doi:10.3390/nano12244496)
Supplement: Supplementary file 1 [file nanomaterials-12-04496-s001.zip › nanomaterials-2044553-supplementary.pdf]

nanomaterials

A Facile Strategy toward the Preparation of a High-Performance Polyamide TFC Membrane with a CA/PVDF Support Layer

Feng Liu [1,2], Yanyan Li [3], Lun Han [1], Zhenzhen Xu [1], Yuqi Zhou [4], Bingyao Deng [4,*] and Jian Xing [1,*]

[1] School of Textile and Garment, Anhui Polytechnic University, Wuhu 241000, China
[2] Advanced Fiber Materials Engineering Research Center of Anhui Province, Anhui Polytechnic University, Wuhu 241000, China
[3] College of Materials and Textile Engineering, Jiaxing University, Jiaxing 314001, China
[4] Laboratory for Advanced Nonwoven Technology, Key Laboratory of Eco-Textiles, Ministry of Education, Jiangnan University, Wuxi 214122, China
Correspondence: bydeng@jiangnan.edu.cn (B.D.); xingjian@ahpu.edu.cn (J.X.); Tel.: +86-13806185561 (B.D.)

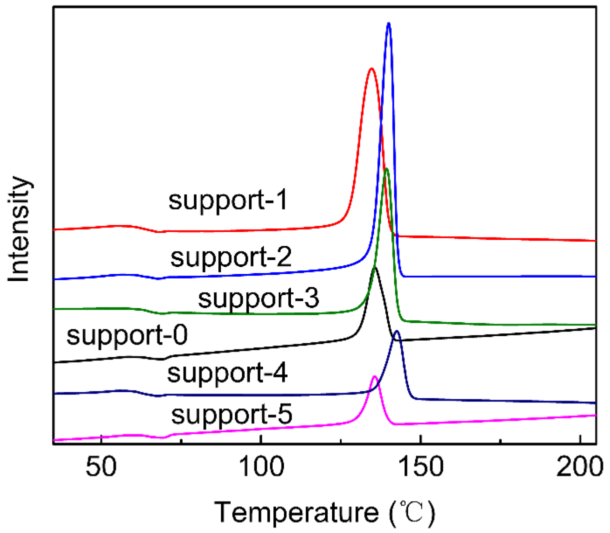

Figure S1. The DSC temperature-rising curves of different supports.

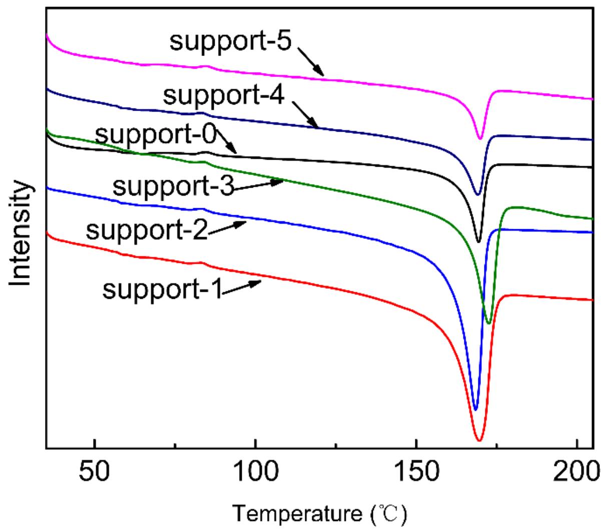

Figure S2. The DSC lowing temperature curves of different supports.

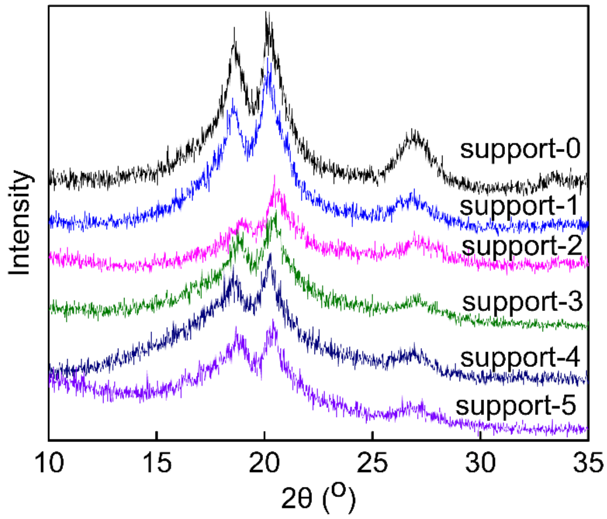

Figure S3. The XRD curves of different supports.

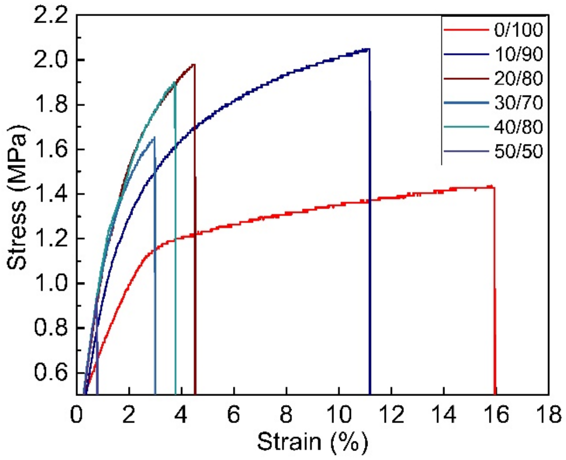

Figure S4. The stress-strain curves of different supports.
